# Central Positional Vertigo at the Onset of Autoimmune Encephalitis Associated With Antibodies to GAD65

**DOI:** 10.7759/cureus.77355

**Published:** 2025-01-12

**Authors:** Adilia Maiylova, Shikhmirza Nabiev, Elena Popova, Olga Kosivtsova, Olga Voskresenskaya

**Affiliations:** 1 Department of Nervous Diseases and Neurosurgery, Faculty of Therapeutics, A.Ya. Kozhevnikov Clinic of Nervous System Diseases, University Clinical Hospital No. 3, I.M. Sechenov First Moscow State Medical University, Moscow, RUS

**Keywords:** anti-gad65 encephalitis, anti-gad antibodies, autoimmune encephalitis, central positional vertigo, neuroimmunology

## Abstract

Vertigo is a common symptom encountered by neurologists in clinical practice. Position-dependent vertigo can occur at the onset of autoimmune encephalitis, often being misinterpreted as benign paroxysmal positional vertigo (BPPV). This study describes a clinical case of a patient with autoimmune encephalitis associated with GAD65 antibodies, who initially presented with systemic positional vertigo.

## Introduction

Autoimmune encephalitis (AE) is an expanding spectrum of neurological disorders characterized by immune-mediated damage to the central nervous system (CNS). In some AE subtypes, antibodies target intracellular or extracellular antigens on CNS cells, leading to neurological dysfunction. AE encompasses paraneoplastic syndromes associated with underlying malignancies and idiopathic cases without neoplasms [1,2].

Diagnosing AE can be challenging due to its diverse clinical presentations, frequent psychiatric symptoms that may mimic primary mental illnesses, lack of MRI abnormalities, nonspecific electroencephalogram (EEG) findings, and absence of identifiable antibodies. However, timely diagnosis is crucial, as early initiation of immunotherapy is most beneficial for patient outcomes [3]. Here, we present a case of an AE with the rare clinical manifestation of central position-dependent vertigo.

## Case presentation

A 51-year-old male patient with autoimmune thyroiditis on L-thyroxine presented with worsening horizontal position-dependent vertigo, gait instability, and hiccups. In February 2022, he developed severe dizziness relieved in an upright position, with a normal brain MRI. Over the following months, he experienced fluctuating dizziness and ataxia. By June 2022, dizziness worsened when lying on the left side, accompanied by intermittent hiccups. A neurological evaluation in September 2022 revealed cerebellar ataxia. A neurological evaluation in September 2022 revealed cerebellar ataxia. The patient’s medical history includes chronic Hashimoto's thyroiditis, diagnosed in 2018, for which he has been taking L-thyroxine (100 mcg daily). A prior instance of elevated thyroid peroxidase antibodies was noted, but the antibody titer was normal upon admission.

On examination at our institution in January 2023, the patient had position-dependent dizziness and gait ataxia. Detailed oculomotor testing revealed vertical nystagmus with a fast downward component when looking to the right. When looking down, the vertical nystagmus was small-amplitude and non-regressive, with no corrective eye movements. The Dix-Hallpike test showed a significant increase in vertical nystagmus, without a latent period, which slowly faded but did not completely regress.

Laboratory workup showed normal vitamin B12, negative anti-gliadin, anti-double-stranded DNA antibodies, antinuclear antibodies (ANAs), antineutrophil cytoplasmic antibodies (ANCAs), and low-titer anti-Ri autoantibodies (ANNA2). CSF analysis revealed no pleocytosis, normal protein, and normal glucose levels. A repeat brain MRI was unremarkable.

Given the mild course atypical for anti-Ri AE, antibody testing was repeated, revealing high-titer anti-GAD65 antibodies in serum (>10,000 IU/mL) and CSF (971 IU/mL; normal, <5 IU/mL), confirming the diagnosis of anti-GAD65 AE.

To exclude an underlying malignancy, CT and PET-CT scans of the chest, abdomen, and pelvis were performed, revealing no neoplasms. Scrotum ultrasound identified a cyst in the right testicular epididymis, and thyroid ultrasound showed no significant pathology.

The patient received pulse methylprednisolone (total 5000 mg), resulting in improvement of hiccups but persistent cerebellar signs and central positional nystagmus. Due to the limited response, a single infusion of 1000 mg cyclophosphamide was administered. Subsequently, the patient reported reduced dizziness and ataxia, with near-complete regression of cerebellar symptoms on neurological examination.

The patient was discharged on a maintenance regimen of methylprednisolone (60 mg for two months) and monthly cyclophosphamide infusions (1000 mg) for six months to prevent recurrent exacerbations.

## Discussion

GAD65 is an intracellular enzyme that converts glutamate to GABA, the main inhibitory neurotransmitter in the CNS. Anti-GAD65 antibodies are associated with various neurological syndromes, including limbic encephalitis, stiff-person syndrome, cerebellar ataxia, and rhombencephalitis [4].

Elevated concentrations of GAD65 antibodies may be associated with other autoimmune conditions, such as type 1 diabetes mellitus, autoimmune thyroiditis, autoimmune polyendocrinopathy, pernicious anemia, and vitiligo [5].

Anti-GAD65 is associated with a low risk of tumor processes. The most common malignant neoplasms associated with it include small-cell lung cancer, neuroendocrine tumors, and malignant thymoma [6]. Paraneoplastic manifestations most often occur in older men with atypical clinical presentations and co-expression of other antineuronal antibodies, as observed in the present case. The phenotypic variant of cerebellar ataxia identified in our patient is seen in 72%-92% of cases with other autoimmune diseases, mainly insulin-dependent diabetes mellitus, pernicious anemia, and autoimmune thyroiditis [7].

The unique feature in this case was the initial presentation of central position-dependent vertigo, which mimicked BPPV. Unlike BPPV, the patient's nystagmus lacked latency, rotatory components, and fatigability. The vertical nystagmus with a fast downward component, which worsened with rightward gaze and the Dix-Hallpike maneuver, indicated damage to the vestibular pathways in the brainstem, cerebellum, or cortex [8,9]. Anti-GAD65 AE can cause ataxia with position-dependent horizontal, upward, or downward nystagmus, likely due to immune-mediated injury to cerebellar Purkinje neurons and vestibular connections [10,11].

Considering the single increase in the titer of antibodies to thyroid peroxidase, a differential diagnosis was conducted for steroid-responsive encephalopathy associated with autoimmune thyroiditis (SREAT), also known as Hashimoto's encephalopathy. However, Hashimoto's encephalopathy presents with a range of clinical symptoms not seen in our patient, such as seizures and disturbances of consciousness in 50% of cases and cognitive impairment and memory decline in 50% of cases. Myoclonus, psychotic syndromes, stroke-like episodes, tremors, involuntary movements, speech disorders, and ataxia are less frequently observed. Laboratory studies confirm the presence of antibodies to anti-thyroid peroxidase and anti-thyroglobulin, but their titer does not correlate with disease activity. The diagnosis is also made in the absence of neuronal antibodies in the serum and cerebrospinal fluid while excluding alternative causes. One of the important diagnostic criteria is a positive response to corticosteroid therapy [1]. However, our patient had a limited response to corticosteroids.

## Conclusions

In summary, this case highlights the diagnostic challenges of anti-GAD65 AE, which can present atypically with central position-dependent vertigo. A high index of suspicion is needed, as early immunotherapy is crucial for optimal outcomes. An extensive diagnostic workup, including repeat antibody testing and malignancy screening, may be necessary to establish the diagnosis.
